# Occurrence of SARS-CoV-2 reinfections at regular intervals in Ecuador

**DOI:** 10.3389/fcimb.2022.951383

**Published:** 2022-09-09

**Authors:** Rommel Guevara, Belén Prado-Vivar, Sully Márquez, Erika B. Muñoz, Mateo Carvajal, Juan José Guadalupe, Mónica Becerra-Wong, Stefanie Proaño, Rosa Bayas-Rea, Josefina Coloma, Michelle Grunauer, Gabriel Trueba, Patricio Rojas-Silva, Verónica Barragán, Paúl Cárdenas

**Affiliations:** ^1^ Instituto de Microbiología, Universidad San Francisco de Quito USFQ, Quito, Ecuador; ^2^ Laboratorio de Biotecnología Vegetal, Universidad San Francisco de Quito USFQ, Quito, Ecuador; ^3^ Division of Infectious Diseases and Vaccinology, School of Public Health, University of California Berkeley, Berkeley, CA, United States; ^4^ Escuela de Medicina, Universidad San Francisco de Quito USFQ, Quito, Ecuador

**Keywords:** COVID-19, reinfection, genomic epidemiology, sequencing, SARS-CoV-2

## Abstract

SARS-CoV-2 reinfection is defined as a new infection with a different virus variant in an individual who has already recovered from a previous episode of COVID-19. The first case of reinfection in the world was described in August 2020, since then, reinfections have increased over time and their incidence has fluctuated with specific SARS-CoV-2 variant waves. Initially, reinfections were estimated to represent less than 1% of total COVID-19 infections. With the advent of the Omicron variant, reinfections became more frequent, representing up to 10% of cases (based on data from developed countries). The frequency of reinfections in Latin America has been scarcely reported. The current study shows that in Ecuador, the frequency of reinfections has increased 10-fold following the introduction of Omicron, after 22 months of surveillance in a single center of COVID-19 diagnostics. Suspected reinfections were identified retrospectively from a database of RT-qPCR-positive patients. Cases were confirmed by sequencing viral genomes from the first and second infections using the ONT MinION platform. Monthly surveillance showed that the main incidence peaks of reinfections were reached within four to five months, coinciding with the increase of COVID-19 cases in the country, suggesting that the emergence of reinfections is related to higher exposure to the virus during outbreaks. This study performed the longest monitoring of SARS-CoV-2 reinfections, showing an occurrence at regular intervals of 4-5 months and confirming a greater propensity of Omicron to cause reinfections.

## Introduction

The outbreak of COVID-19 was declared a public health emergency of international concern in March 2020 by the World Health Organization (WHO), more than two years later the pandemic has not been fully controlled as emerging variants keep producing epidemic waves (Wise, 2022). The high evolutive rate of SARS-CoV-2 (Tay et al., 2022) contributes to the emergence of fitter variants with increased transmissibility and greater ability to partially evade immune protection acquired through natural infection or vaccination (Hu et al., 2022). Given that around half of the world’s population is estimated to have been infected with COVID-19 at least once (Barber et al., 2022), the next epidemic waves are expected to be caused primarily by reinfections. SARS-CoV-2 reinfection is defined as a new infection with a different viral variant in individuals who have already recovered from a previous episode of COVID-19 (Yahav et al., 2021). The lack of sequencing technologies to confirm reinfections, mainly in low-income countries, could mask the real impact of reinfections globally, making it difficult to elucidate its risk factors and ways to prevent it in the face of future variants.

The first case of SARS-CoV-2 reinfection sequence-confirmed was reported in Hong Kong in August 2020 (To et al., 2020) and since then several cases were reported worldwide leading to scientific efforts to reinfection scrutiny. Multiple protocols were proposed to identify suspected cases and to rule out the persistence or relapse of early infecting variants. The Pan American Health Organization (PAHO) defined a suspected case of SARS-CoV-2 reinfection as a symptomatic individual with a positive test after 45 days since the prior infection or 90 days if was asymptomatic, and suggested the confirmation of the case based on laboratory evidence (sequencing of both viral variants) or epidemiological data (negative test between events) (PAHO, 2020). Early estimates from the USA, UK, Denmark, and Qatar reported a low frequency of SARS-CoV-2 reinfections, accounting for less than 1% of total infections (Abu-Raddad et al., 2021; Hall et al., 2021; Hansen et al., 2021; Harvey et al., 2021). After the arrival of the Omicron variant in late 2021, the frequency of reinfections increased to about 10% of total infections (Bastard et al., 2022; Pulliam et al., 2022). Nevertheless, these proportions were mainly estimated in high-income countries and the situation in other countries remained elusive.

Latin America was one of the most affected regions during the COVID-19 pandemic, mainly driven by a deficient public health system coupled with socioeconomic problems in its population (Bakker, 2021). The first confirmed case of SARS-CoV-2 reinfection in this region was reported in July 2020 in Ecuador (Prado-Vivar et al., 2021) followed by other reports in different countries (Díaz et al., 2021; Fonseca et al., 2021; Ramírez et al., 2021). However, most of these reports were isolated cases that do not allow elucidation of the frequency of reinfections and the impact of emerging variants. This study describes SARS-CoV-2 reinfection cases identified retrospectively in Ecuador after 22 months of surveillance. Suspected cases were identified based on the PAHO guidelines (PAHO, 2020) with some modifications to include possible reinfections occurring in less than 45 days, meanwhile, the confirmation of the case was done by sequencing SARS-CoV-2 genomes from the first and second infection using the ONT MinION platform. This study performs the most extended follow-up of SARS-CoV-2 reinfections in a low-income country and contributes to the global effort of genomic surveillance of emerging variants that could help clarify the leading causes of reinfections and understand the dynamics of COVID-19 epidemic waves.

## Materials and methods

### Sample collection and diagnostics

Samples included in the study were collected from clinics and hospitals in different provinces of Ecuador and tested at the diagnostic center of the Microbiology Institute of Universidad San Francisco de Quito (IM-USFQ) from May 2020 to February 2022. Demographic and clinical information for each patient was retrieved at the same time as sample collection and was derived to IM-USFQ. Specimens were oropharyngeal, or nasopharyngeal swabs taken from symptomatic and asymptomatic patients. Swabs were collected in a 1.5 mL sterile tube with 1X DNA/RNA Shield (Zymo, USA) which inactivates the virus and preserves its genomic material. Samples were immediately transported to the laboratory at room temperature in a sealed container. The diagnosis of SARS-CoV-2 was based on RT-qPCR using one of the following kits: the Veri-Q PCR 316 kit (MiCo BioMed, South Korea), the LightMix^®^ SarbecoV E-gene kit (TIB Molbiol, Germany), or the Allplex™ 2019-nCoV Assay (Seegene, South Korea). The cycle threshold (Ct) value to identify positive results was based on manufacturers’ instructions. Positive samples were sequenced as described below.

### Selection of patients and ethical considerations

**Figure 1:**
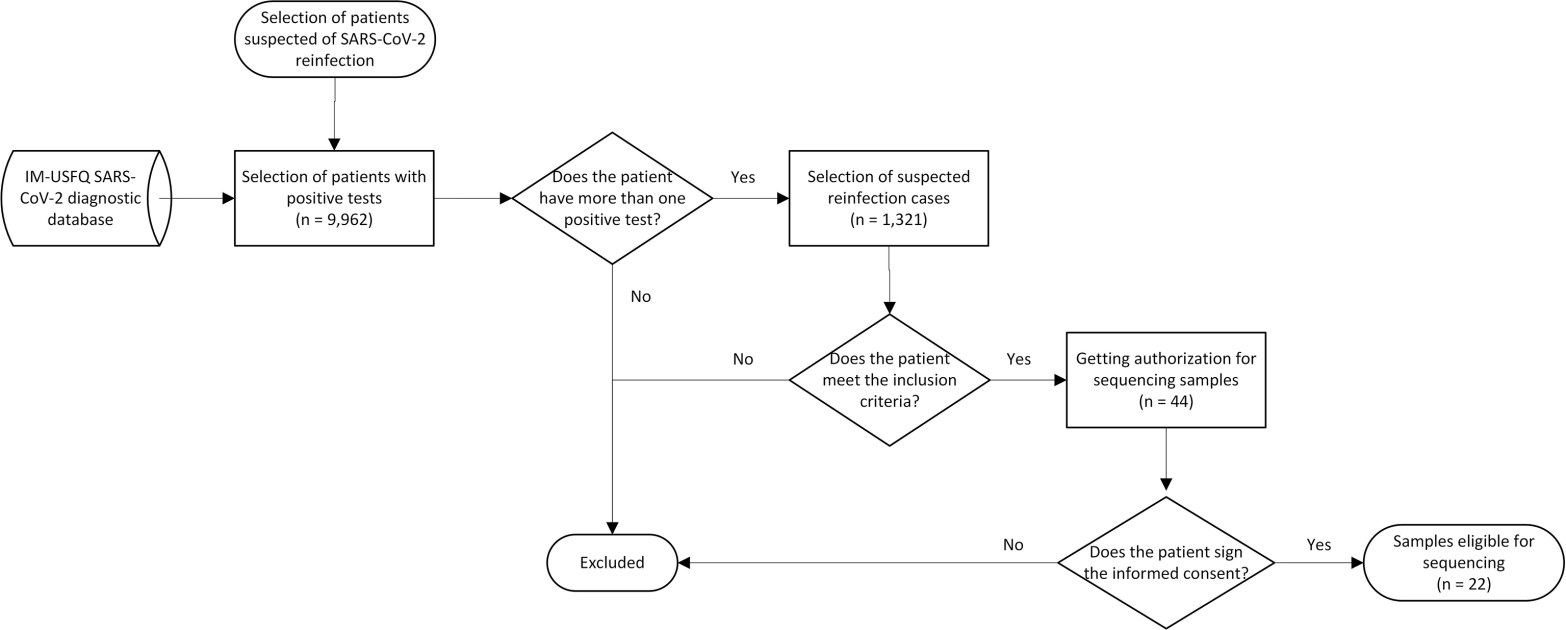
Flow chart for the selection of suspected reinfections. The database includes tests performed at IM-USFQ from May 2020 to February 2022. Suspected cases were identified retrospectively, first by patients who reported more than one positive test, and then by the compliance with inclusion criteria: at least 45 days between infections, or less if the Ct revealed a higher viral load in the reinfection. From the 44 patients who accomplished inclusion criteria, 22 were considered for sequencing.

Identification of suspected reinfections was performed retrospectively and based on PAHO recommendations (PAHO, 2020) with some modifications. The process is represented in a flow chart in **Figure 1**. The IM-USFQ database of RT-qPCR test was used, which compiled every test processed by this diagnostic center since May 2020. Patients who have more than one positive test were selected and defined as suspected reinfection based on inclusion criteria that consider people with an interval of more than 45 days between infections, and to prevent exclusion of reinfections occurring in less time, were included those cases where the second test showed evidence of higher viral loads by a lower Ct-value than the first test. This study also includes a reinfected patient previously reported by the IM-USFQ (Prado-Vivar et al., 2021). Patients suspected of reinfection were reached to get their informed consent to sequence their samples and include their data in the study, which was managed anonymously through the assignation of identification codes. Demographic data were included, as well as the available clinical records, which differed for each patient depending on the care center of origin. Symptoms were classified as mild, moderate, or severe according to the WHO criteria (WHO, 2020). This study is part of a country-wide project that aims to monitor the SARS-CoV-2 variants in Ecuador which were approved by the Bioethics Committee of Universidad San Francisco de Quito (CEISH No. P2020-022IN) and by the Ministerio de Salud del Ecuador (MSP-CGDES-2020-0121-O).

### SARS-CoV-2 whole-genome sequencing

The RNA extraction, cDNA preparation, and sequencing were done as described previously by (Prado-Vivar et al., 2021). Briefly, the Quick RNA viral w/zymo-Spin IC (Zymo, USA) kit was used for RNA extraction. Retro-transcription to cDNA was carried out in line with the ARTIC network protocol (Quick, 2020). The cDNA obtained was stored at 4°C for the next step. A long-read sequencing approach through MinION™ (Oxford Nanopore Technologies, UK) was used following the ARTIC network protocol (Quick, 2020) specific for SARS-CoV-2 sequencing. In brief, target enrichment was performed using multiplex-PCR, with primer schemes V1 and V3 (Quick, 2020; Tyson et al., 2020), over the cDNA previously prepared. Amplification was assessed by agarose gel electrophoresis and the product was quantified using the Qubit dsDNA HS assay kit (Invitrogen, USA). After normalization, the library was prepared following manufacturer instructions by using the Native Barcoding Kit (EXP-NBD196) with the Ligation Sequencing Kit (SQK-LSK-109). The genomic library was loaded into a MinION flow cell (FLO-MIN106D). The sequencing was programmed in the software MinKNOW v22.03.4 with fast basecalling, demultiplexing, and adapter removal enabled. The real-time monitoring of the sequencing process was carried out using the RAMPART software v1.2.0 (Rambaut, 2020). After sequencing, the Medaka v1.6.0 algorithm was used for variant calling and to create consensus sequences mapped against the Wuhan-Hu-1 reference genome (NC_045512.2). Consensus sequence quality was assessed by Nextclade v1.14.0 parameters (clades.nextstrain.org) and were uploaded to the GISAID repository (gisaid.org).

### Data and phylogenetic analysis

Clades and linages assignments were performed using Nextclade and Pangolin COVID-19 Lineage Assigner v4.0.5 (pangolin.cog-uk.io), respectively. Nextclade was used to identify nucleotide mutations and amino acid substitutions on each sequence relative to the reference genome. A phylogenetic tree was built to ensure that paired viral sequences from each reinfection had a different immediate ancestor. The Wuhan-Hu-1 reference genome (GenBank accession: MN908947.3) was included for rooting the phylogram. These sequences were aligned along with reinfection sequences using MAFFT online program (Katoh et al., 2019). Finally, the phylogram was estimated by the Maximum Likelihood method under a GTR nucleotide substitution model and 1000 bootstrap replicates using IQ-TREE online tool (hiv.lanl.gov/content/sequence/IQTREE/iqtree.html). The phylogram figure was obtained and annotated using the iTOL v6.5.2 (Letunic and Bork, 2021). Statistical analysis was done in RStudio v2022.02.1 with the package descriptr (Hebbali, 2020). The confidence interval (CI) of incidences was estimated by Wald’s test with continuity correction. The variation of symptoms and Ct-value between first and second infections were assessed with the McNemar’s Chi-squared test with continuity correction, where symptoms were categorized as moderate or mild/asymptomatic, and Ct-values as < 30 or > 30. A *p*-value < 0.05 was considered statistically significant.

## Results

### Suspected reinfection cases

From May 2020 to February 2022, the IM-USFQ diagnostic database registered 9,962 patients with at least one positive RT-qPCR test for SARS-CoV-2; 1,321 of them (13.26%) had more than one positive sample, and from this subset suspected reinfection cases were selected. 44 patients (0.44% [95% CI 0.32-0.60]) met the inclusion criteria (**Figure 1**) and their demographic data is shown in **Table S1**. 28 (64%) were male patients, their average age was 36 years old (median 34.5; range 19-57), and the mean interval between infections was 237 days (median 226; IQR 90-318). 15 patients (34%) were not vaccinated during reinfection, whereas five (11%) had the first dose, 19 (43%) had the second dose, and four (9%) had the booster dose. Most patients were from Quito, the capital city of Ecuador, eight were from Manta in the Coastal region, and two were from the Amazon region (Tena and Nueva Loja). Clinical records were not available for all patients, however, none had severe symptomatology in either the first and second infection, and 13 out 25 reported milder or any symptoms during reinfection. Neither the variation of symptoms and Ct-values between the first and second reinfection were considered statistically significant (*p* > 0.05).

**Figure 2:**
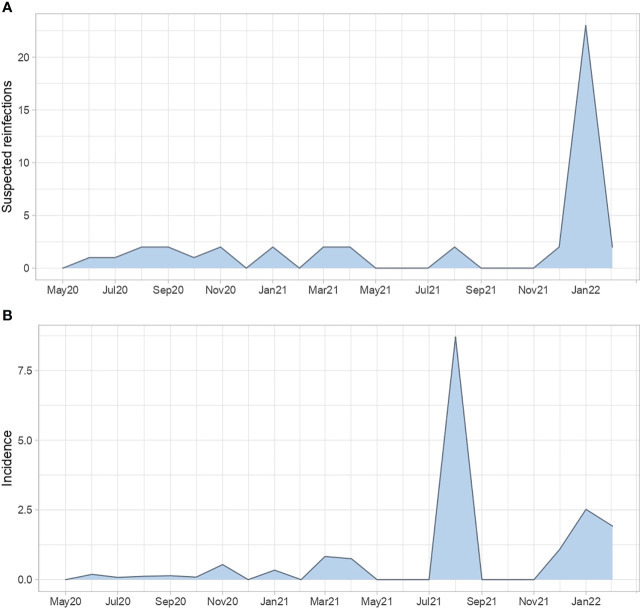
Monthly surveillance of reinfections. Suspected cases identified each month within the 22 months period **(A)** and the corresponding incidence regarding the total SARS-CoV-2 positive tests **(B)**.

**Figure 2** shows suspected reinfections identified during each month of surveillance. The first case was identified in June 2020, whereas the latter was in February 2022 (panel A), detecting at least one case in 13 out of the 22 months of surveillance. The figure also shows the proportion of suspected reinfections in relation to the total positive tests processed each month (Panel B), showing the highest point of incidence in August 2021 (8.70%), followed by January 2022 (2.52%), February 2022 (1.92%), and December 2021 (1.08%).

### Genomic sequencing of SARS-CoV-2

**Figure 3:**
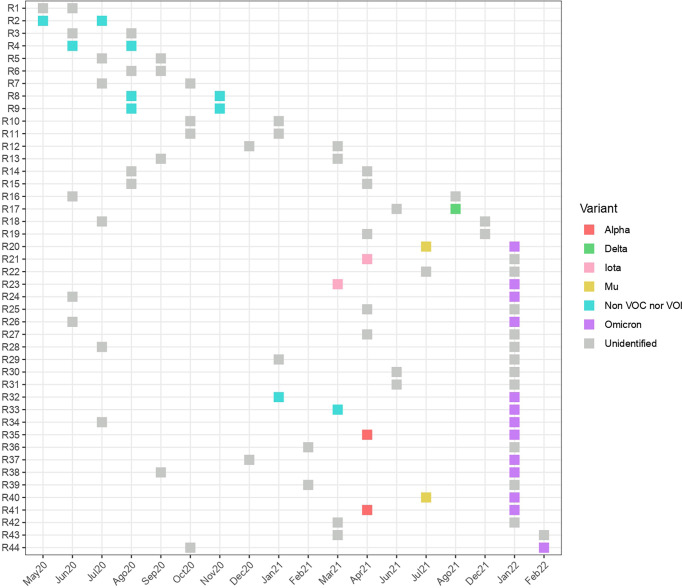
Timing of reinfections and variants identified. Plot of the first and second infection detected for each patient (y-axis) over the 22-month surveillance period (x-axis). Each identified variant is represented in different colors.

**Figure 4:**
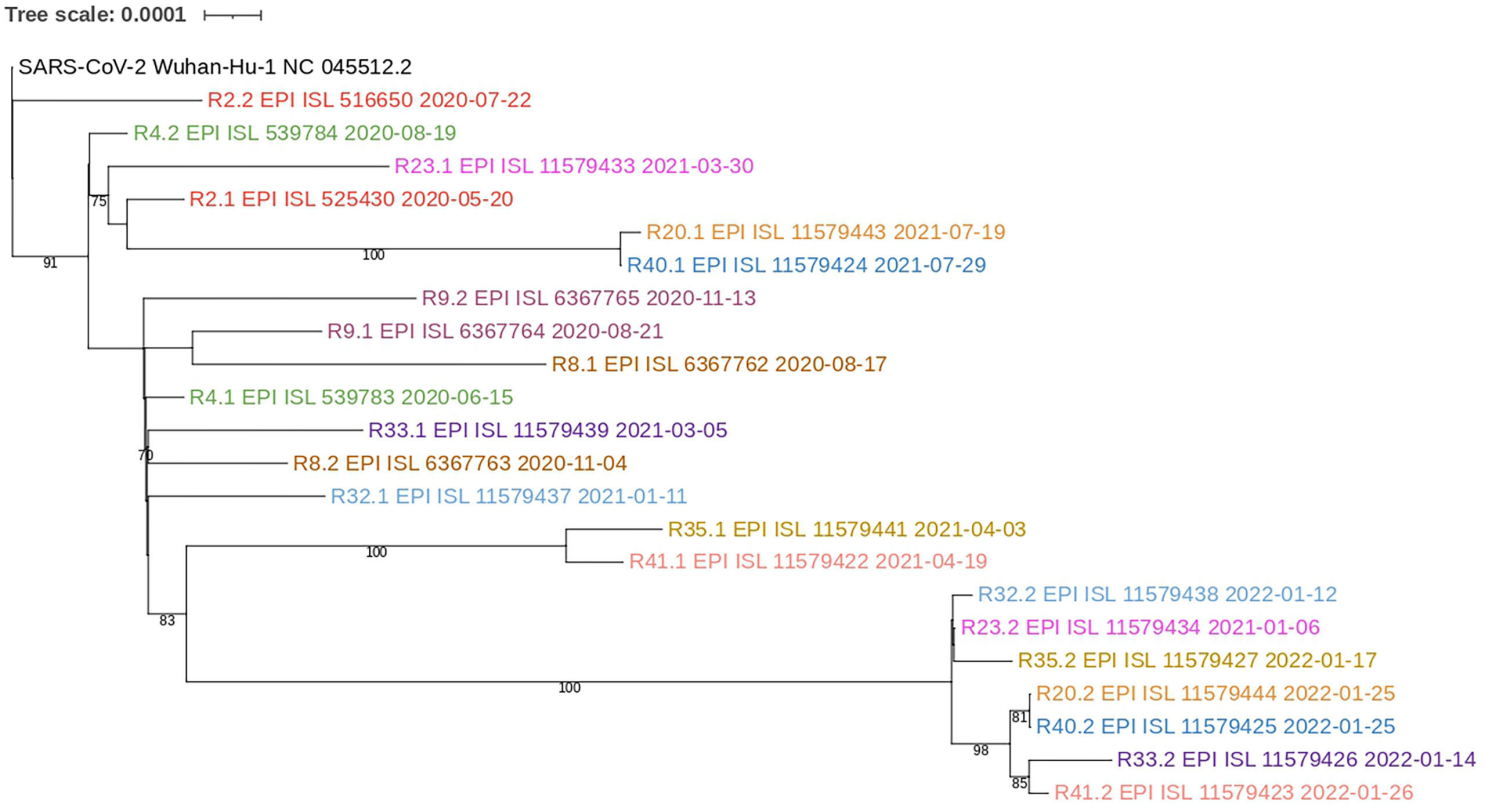
Phylogram of paired sequences. Only paired consensus sequences were used and rooted to the Wuhan-Hu’s SARS-CoV-2 reference genome. Each pair is represented in different colors. Bootstraps equal to or above 70 are shown.

Informed consents were obtained for 22 out of 44 patients suspected of SARS-CoV-2 reinfection (**Figure 1**). Samples from 19 patients were successfully sequenced, including 11 patients with paired samples from their first and second infection, and eight patients only with the second infection sample. Clades and lineages assigned to each genome are shown in **Table S2**, together with amino acid substitutions and the GISAID accession number. Isolates from 2020 were assigned as common variants, whereas variants from 2021 and 2022 were classified as variants of interest (VOI) and variants of concern (VOC) (**Figure 3**). All the reinfecting variants from January and February 2022 were classified as Omicron. The incidence of reinfections during the Omicron period (since December 2021) was 2.45% (95% CI 1.63-3.66), whereas in the pre-Omicron period it was 0.21% (95% CI 0.13-0.34). In the paired samples, the reinfecting virus variant was from a different clade and/or lineage than the first infecting virus variant. This was confirmed by the phylogenetic tree where none of the reinfecting variants shares an immediate ancestor with the first infecting virus variant (**Figure 4**). These results confirm the reinfections of 11 patients out of 44 suspected cases (25%). Only four of them presented more severe symptoms during reinfections, and none reported significant clinical sequelae.

## Discussion

Suspected reinfections of SARS-CoV-2 were retrospectively identified in the period from May 2020 to February 2022, with 11 cases confirmed by sequencing of the infecting variants. The incidence of suspected reinfections increased by 10-fold after the introduction of the Omicron variant. Monthly surveillance showed that the proportion of reinfections detected was higher at regular intervals of four to five months. This study reports a 22 month-period surveillance of SARS-CoV-2 reinfections, where COVID-19 infection was detected by RT-qPCR.

SARS-CoV-2 is an RNA virus with an unexpectedly high rate of evolution (Tay et al., 2022), which has contributed to the continuous emergence of more virulent variants and the surge of several epidemic waves since the onset of the COVID-19 pandemic. The first cases of reinfections by SARS-CoV-2 confirmed the ability of the virus to partially evade the immune protection previously acquired by natural infection, as has been seen with other closely related viruses (Edridge et al., 2020). Early reports estimated a low frequency of reinfections (less than 0.70%) regarding to the total of newly infected individuals (Abu-Raddad et al., 2021; Hall et al., 2021; Hansen et al., 2021; Harvey et al., 2021), however, based on short periods of surveillance and lacking confirmation of reinfection by sequencing approaches. After 20 months of follow-up (during the pre-Omicron period), our study confirms the low rate of reinfections, with an incidence of 0.21% of suspected cases and only four events confirmed by sequencing, were any reinfecting variant shared an immediate ancestor with the prior infecting variant (**Figure 4**). Nevertheless, the arrival of the Omicron variant marked a turning point regarding the frequency of reinfections detected, not seen before for other VOCs, with a 10-fold increase in the proportion of cases (Bastard et al., 2022; Nunes et al., 2022; Pulliam et al., 2022). This characteristic of Omicron is attributed to the more than 30 mutations identified in this variant, mainly in the RBD of the Spike protein and together with insertions and deletions (Akkız, 2022), that confer an increased ability to evade immune protection, including that acquired by vaccination (Bazargan et al., 2022). As expected, the incidence of suspected reinfections detected in Ecuador in this study increased to 2.45% during the Omicron period, and sequencing confirmed that the reinfecting variants were from this clade (**Figure 3**). Although not all samples could be sequenced, it is expected that they were also Omicron, considering that by early January 2022 this was the dominant variant in Ecuador (covariants.org/per-country). Despite we were able to only confirm 11 reinfections out of 44 suspected cases other events could be misdiagnosed, because is estimated that half of COVID-19 infections remains asymptomatic (Cohen et al., 2022).

The COVID-19 pandemic has been evolving dynamically due to the surge of multiple waves of infections related to the emergence of more virulent variants of SARS-CoV-2. South America has been one of the most affected regions in the world, driven by a precarious healthcare system and aggravated by the late arrival of vaccines. The first case in Ecuador was reported in February 2020, followed by the surge of the first wave of infections in April 2020, with the city of Guayaquil, the country’s main port, as the first epicenter of the pandemic (Gutierrez et al., 2021). Quito, the capital city of Ecuador, overtook Guayaquil as the epicenter of cases between June and July 2020, and precisely at that time the IM-USFQ detected the first suspected case of SARS-CoV-2 reinfection (**Figure 2**). Since then, we observed an increase in the rate of cases in November 2020 (0.54%), April 2021 (0.75%), August 2021 (8.70%), and January 2022 (2.52%) (**Figure 2**). All of these months were identified as periods of ongoing waves of infections in the country, possibly driven by the relaxation of biosafety measures during holidays such as the Day of the Dead and the Holy Week celebrated in November and April, respectively, or by the surge of fitter variants such as Delta and Omicron during August 2021 and January 2022, respectively (MSP, 2022; MSP, 2021). This evidence suggests that the increase in the rate of suspected reinfections may be driven by waves of infections, where augmented exposure to SARS-CoV-2 could increase the risk of becoming reinfected. Our results further show the occurrence of the highest frequencies of suspected reinfections at regular intervals of four to five months (**Figure 2**), in agreement with the dynamics observed for the emergence of waves of infections in different countries during the COVID-19 pandemic (Callaway, 2022). This predictable behavior may be useful to prevent future surges of infections considering that at least half of the world’s population has already been infected by SARS-CoV-2 (Barber et al., 2022) but remains susceptible to reinfections.

A waning immunity, either naturally induced or vaccine-induced, could also explain the occurrence of higher reinfection rates on a regular basis. Early reports suggested that the duration of protection from naturally induced immunity could be at least six months (Biobank UK, 2021; Lumley et al., 2021), close to the 5-month interval observed in this study between the highest rates of suspected reinfections in unvaccinated population (before May 2021) (**Figure 2**). On the other hand, the application of vaccines strengthened the immune response in both naïve and convalescent individuals, inducing in the latter the so-called “hybrid immunity” that is expected to last longer (Pilz et al., 2022). In Ecuador, the vaccination program started late, immunizing most of the population since May 2021. This study identified suspected reinfections in vaccinated individuals since August 2021 (**Table S1**), but in persons with half-basic vaccination schemes, and subsequently most cases were in persons with basic schemes completed four months prior to reinfection. This evidence confirms the vulnerability of individuals without completed vaccination schemes and boosters, and suggests a shorter duration of the hybrid immunity, however, as we did not measure the seropositivity of reinfected patients, more evidence is needed.

As SARS-CoV-2 reinfections continue to occur, questions arise about the consequences of this event. Recent evidence suggests that reinfections increase the risk of death, hospitalization, and negative effects in the pulmonary and extrapulmonary systems, proportionally depending on the number of reinfections in the patient (Al-Aly et al., 2022). This reinforces the need of reinfections monitoring, which has been done according to different guidelines proposed by the PAHO, the Center for Disease Control and Prevention (CDC, 2020), and the European CDC (ECDC, 2021), all of them agreeing that most reinfections occur after 60 days since the prior infection (CDC, 2020; PAHO, 2020; ECDC, 2021). Nevertheless, there are reports of sequence-confirmed reinfections by common variants occurring in less than 45 days (Lee et al., 2020; Rani et al., 2021; Tillett et al., 2021) and in less than 60 days by the Omicron variant, mainly in young and unvaccinated individuals (Nevejan et al., 2022). Our inclusion criteria included cases with intervals of 45 days or less if there was evidence of increased viral load (lower Ct-value), however, only one case was identified with an interval of 34 days that could not be confirmed by sequencing (**Table S1**). In fact, most of the suspected cases occurred after a 90-days interval, in agreement with the guidelines mentioned before. Other parameters could be useful for the identification reinfections, such as variation in symptoms and Ct-values between first and second infections. The latter was proposed by other studies showing a significant difference in Ct-values between paired samples (Ringlander et al., 2021) and supporting its use as marker to differentiate reinfections from persistence (Julian et al., 2021). Nevertheless, our results show no significant variation in either symptoms or Ct-values and cannot support their use as markers of reinfections, even because Ct-values depends on external factors such as the time elapsed since the onset of symptoms. Despite the lack in the clinical records of all patients, none reported severe symptomology during reinfection, including those sequence-confirmed cases, which was expected due to the immunity protection acquired after the prior infection and reinforced by vaccination. However, more evidence is needed to confirm these outcomes in other age groups more susceptible to severe illness and sequelae.

This study has some limitations. Since the IM-USFQ mainly received samples from Quito, with less representation of other cities in Ecuador, results cannot be extrapolated to the whole country. Also, results did not reflect the situation of the whole population because our database was mainly composed of immunocompetent adults and working individuals. We lack the complete clinical records of surveilled patients, as this information was collected by other institutions. Additionally, we did not confirm all reinfections by sequencing because we cannot reach some patients or we did not recover enough RNA in their samples, possibly due to low viral titers. Finally, we were not able to characterize the neutralizing antibodies in patients, which could have been useful to explain reinfections.

## Conclusion

This study reports an increased frequency of reinfections in Ecuador after the introduction of the Omicron variant and within 22 months of surveillance. The highest incidence of suspected cases was reached at regular intervals of four to five months and during ongoing outbreaks. Most cases occurred in male adults without a booster dose of the vaccine and after 90 days since the prior infection. These results show a regular dynamic in the occurrence of reinfections that could be useful in preventing future reinfections by emerging variants.

## Data availability statement

The data presented in this study are deposited in the GISAID repository, and the accession numbers are found in **Table S2** of **Supplementary material**.

## Ethics statement

This study is part of a country-wide project that aims to monitor the SARS-CoV-2 variants in Ecuador which were approved by the Bioethics Committee of Universidad San Francisco de Quito (CEISH No. P2020-022IN) and by the Ministerio de Salud del Ecuador (MSP-CGDES-2020-0121-O). The patients/participants provided their written informed consent to participate in this study.

## Author contributions

Conceptualization: VB and PC; methodology: RG, VB, and PC; laboratory work: RG, BP-V, SM, JG, EM, MC, MB, SP, and RB-R; software: RG, EM, MC, BP-V, and PC; analysis: RG, VB, PC, GT, and PR-S; writing – original draft: RG; writing – review and editing: VB, PC, GT, PR-S, EM, MC, and MJC; funding acquisition: PC, MG, and MJC. All authors contributed to the article and approved the submitted version.

## Funding

This study was funded by the NIH supplement grant U01AI151788 through A2CARES-CREID. “RED PARA ELDESARROLLO DE INSTRUMENTOS INNOVADORES APLICADOS A LA INVESTIGACIÓN EPIDEMIOLÓ GICAEN AMÉRICA DEL SUR” through the Government of France and Instituto Pasteur Montevideo. RISE-USAID Project Ecuador.

## Acknowledgments

We would like to thank the SIME hospital at USFQ and the other healthcare centers that collected samples and provided patients’ data. We are thankful for the support of the SARS-CoV-2 diagnostic team at IM-USFQ. We are also grateful to the developers and contributors of open-access databases and free online bioinformatic tools.

## Conflict of interest

The authors declare that the research was conducted in the absence of any commercial or financial relationships that could be construed as a potential conflict of interest.

## Publisher’s note

All claims expressed in this article are solely those of the authors and do not necessarily represent those of their affiliated organizations, or those of the publisher, the editors and the reviewers. Any product that may be evaluated in this article, or claim that may be made by its manufacturer, is not guaranteed or endorsed by the publisher.
